# Vitamin K_2_ Biosynthetic Enzyme, UBIAD1 Is Essential for Embryonic Development of Mice

**DOI:** 10.1371/journal.pone.0104078

**Published:** 2014-08-15

**Authors:** Kimie Nakagawa, Natsumi Sawada, Yoshihisa Hirota, Yuri Uchino, Yoshitomo Suhara, Tomoka Hasegawa, Norio Amizuka, Tadashi Okamoto, Naoko Tsugawa, Maya Kamao, Nobuaki Funahashi, Toshio Okano

**Affiliations:** 1 Department of Hygienic Sciences, Kobe Pharmaceutical University, Kobe, Japan; 2 Department of Bioscience and Engineering, Shibaura Institute of Technology, Saitama, Japan; 3 Developmental Biology of Hard Tissue, Division of Oral Health Science, Hokkaido University Graduate School of Dental Medicine, Hokkaido, Japan; 4 Department of Health Sciences and Social Pharmacy, Faculty of Pharmaceutical Sciences, Kobe Gakuin University, Kobe, Japan; Nihon University School of Medicine, Japan

## Abstract

UbiA prenyltransferase domain containing 1 (UBIAD1) is a novel vitamin K_2_ biosynthetic enzyme screened and identified from the human genome database. UBIAD1 has recently been shown to catalyse the biosynthesis of Coenzyme Q10 (CoQ10) in zebrafish and human cells. To investigate the function of UBIAD1 in vivo, we attempted to generate mice lacking *Ubiad1*, a homolog of human *UBIAD1*, by gene targeting. *Ubiad1*-deficient (*Ubiad1* ^−/−^) mouse embryos failed to survive beyond embryonic day 7.5, exhibiting small-sized body and gastrulation arrest. *Ubiad1* ^−/−^ embryonic stem (ES) cells failed to synthesize vitamin K_2_ but were able to synthesize CoQ9, similar to wild-type ES cells. *Ubiad1* ^+/−^ mice developed normally, exhibiting normal growth and fertility. Vitamin K_2_ tissue levels and synthesis activity were approximately half of those in the wild-type, whereas CoQ9 tissue levels and synthesis activity were similar to those in the wild-type. Similarly, UBIAD1 expression and vitamin K_2_ synthesis activity of mouse embryonic fibroblasts prepared from *Ubiad1* ^+/−^ E15.5 embryos were approximately half of those in the wild-type, whereas CoQ9 levels and synthesis activity were similar to those in the wild-type. *Ubiad1* ^−/−^ mouse embryos failed to be rescued, but their embryonic lifespans were extended to term by oral administration of MK-4 or CoQ10 to pregnant *Ubiad1* ^+/−^ mice. These results suggest that UBIAD1 is responsible for vitamin K_2_ synthesis but may not be responsible for CoQ9 synthesis in mice. We propose that UBIAD1 plays a pivotal role in embryonic development by synthesizing vitamin K_2_, but may have additional functions beyond the biosynthesis of vitamin K_2_.

## Introduction

Vitamin K is a cofactor for gamma-glutamyl carboxylase (GGCX), an enzyme that converts specific glutamic acid residues in several substrate proteins involved in blood coagulation and bone metabolism to gamma-carboxyglutamic acid (Gla) residues [1], [2]. To date, 19 Gla-containing proteins have been found in vertebrates. Besides its role as a cofactor for GGCX, vitamin K is involved in the transcriptional regulation of the nuclear receptor SXR/PXR [3]–[5] and regulates PKA signalling in osteoblasts and hepatocellular carcinoma cells [6]. Vitamin K functions as a mitochondrial electron carrier during ATP production by the electron transport chain in *Drosophila* [7].

There are two naturally occurring forms of vitamin K, phylloquinone (PK) or vitamin K_1_ and the group of menaquinones (MKs). All forms of vitamin K share a common 2-methyl-1,4-naphthoquinone nucleus, differing from one another in the length and degree of saturation of the aliphatic side chain at the 3 position. PK has a monounsaturated side chain of four isoprenyl residues, and is primarily found in leafy green vegetables. MKs can be classified into 14 types on the basis of the length of their unsaturated side chains. MK-4 or vitamin K_2_ is predominantly present in poultry products, whereas MK-7–MK-10 are exclusively produced by bacteria and gut microflora in mammals. Menadione (MD) or vitamin K_3_ is a synthetic compound that lacks a side chain, although it is believed to be biologically active by virtue of its conversion to MK-4 in the body [8].

Interestingly, dietary PK releases MD by the cleavage of the side chain in the intestine, followed by the delivery of MD via the mesenteric lymphatic system and blood circulation to tissues, where it is converted to MK-4 by the prenylating enzyme UBIAD1 and accumulates in the form of MK-4 [9]. UBIAD1 is a recently identified vitamin K_2_/MK-4 biosynthetic enzyme exhibiting various subcellular localisations including the endothelial reticulum [10], [11], Golgi complex [11], [12] and mitochondria [13] in a variety of tissues and cell types of vertebrates. Whether UBIAD1 has any functions beside the biosynthesis of MK-4 is unknown, but *UBIAD1*/*ubiad1* mutations in zebrafish have been reported to cause cardiac oedema and cranial haemorrhages [12], [14] and *UBIAD1*/*heixuedian (heix)* mutations in *Drosophila* cause defects in mitochondrial ATP production [7]. In humans, mutations in *UBIAD1* cause a rare autosomal-dominant eye disease called Schnyder corneal dystrophy (SCD). SCD is characterised by abnormal deposition of cholesterol and phospholipids in the cornea, resulting in progressive corneal opacification and vision loss [15]. UBIAD1 (also known as transitional epithelial response protein 1 (TERE1)) suppressed the proliferation of transitional cell carcinoma cell lines and prostate cancer cell lines [16]–[20]. However, whether UBIAD1 is involved directly in the above biological responses or indirectly through the biosynthesis of MK-4 remains unknown. It has recently been reported that UBIAD1 catalyses the non-mitochondrial biosynthesis of CoQ10 in zebrafish [12]. Coenzyme Q (CoQ) exists in several forms and can be found in microorganisms, plants and mammals, including humans. CoQ6, Q7 and Q8 are found in yeast and bacteria, whereas CoQ9 is found in rats and mice. CoQ10 is prevalent in humans and zebrafish. CoQ is an endogenously synthesized electron carrier that is critical for electron transfer in the mitochondrial membrane for respiratory chain activity, and as a lipid-soluble antioxidant it plays an important role in protecting biological membranes from oxidative damage. The biosynthesis of CoQ in mitochondria has been studied exclusively in bacteria and yeasts. To investigate the functions of UBIAD1 in vivo, we attempted to generate mice completely lacking *Ubiad1* by gene targeting. We found that *Ubiad1*-deficient (*Ubiad1* ^−/−^) mice uniformly died between embryonic day (E) 7.5 and E10.5 and that *Ubiad1* ^−/−^ mouse embryos failed to be rescued, but their embryonic lifespans were extended partially to term by oral administration of MK-4 or CoQ10 to pregnant *Ubiad1* ^+/−^ mice, indicating that UBIAD1 plays a pivotal role in the embryonic development of mice.

## Materials and Methods

### Materials

Deuterium-labelled MD (MD-d_8_) was purchased from C/D/N Isotopes, Inc. ^13^C-labelled 4-hydroxybenzoate (^13^C_6_-4HB) was purchased from Santa Cruz. PK epoxide, MK-4 epoxide, ^18^O-labelled PK and MK-4 (PK-^18^O and MK-4-^18^O), deuterium-labelled PK and PK epoxide (PK-d_7_ and PK-d_7_ epoxide), deuterium-labelled MK-4 and MK-4 epoxide (MK-4-d_7_ and MK-4-d_7_ epoxide) were synthesized in our laboratory as reported previously [8], [21]. CoQ9 and CoQ10 were donated by Eisai Co. The water-soluble type CoQ10 powder (PureSorb-Q™40. CoQ10 content is 40 w/w% hereafter P40) developed by Nisshin Pharma Inc. (Tokyo, Japan) was used [22].

### Ethics statement

All animal experimental protocols were performed in accordance with the Guidelines for Animal Experiments at Kobe Pharmaceutical University and were approved by The Animal Research and Ethics Committee of Kobe Pharmaceutical University, Kobe Japan. All surgery was performed under sodium pentobarbital anesthesia, and all efforts were made to minimize suffering.

### Generation of *Ubiad1*-deficient mice

pPE7neoW-F2LF, which contains a single loxP site, two flippase recombination target (FRT) sites and a neomycin resistance (neoR) cassette, and pMC-DTA, which contains the diphtheria toxin A gene (DTA), a negatively selective marker, were kindly provided by Dr. K. Yusa (Osaka University, Osaka, Japan) [23]. In this study, pPE7neoW-F2LF was digested with EcoRI and HindIII, and an oligonucleotide linker containing NheI, KpnI and loxP sites was inserted. The resulting plasmid pPE7neoWF2LR/loxP was digested with SacI and SalI to generate a loxP-FRT-neoR-FRT-loxP fragment. The DNA fragment was ligated into the SacI and SalI sites of the pMCS-DTA vector to generate pMCS-DTA-cKO. The *Ubiad1* exon1 was amplified with an SpeI-anchored sense primer (SpeI_DA_F: CCCTGAAATCCCAGGAGGGCTAAACAG) and a KpnI-anchored antisense primer (KpnI_DA_R: CAAAGACGCCTTACTAAAGTAGGCCACTT) from mouse genomic DNA (Clontech Lab., Inc.), and cloned into NheI–KpnI-digested pMCS-DTA-cKO. The *Ubiad1* 5′-flanking region was amplified with a SalI-anchored sense primer (SalI_5A_F: GCTCGTAAGCGCTACAACCAATCAG) and a ClaI-anchored antisense primer (ClaI_5A_R: CCCAGTATAACGCAAAGCGACACG) from mouse genomic DNA and cloned into pMCS-DTA-cKO. The *Ubiad1* 3′-flanking region was amplified with a NotI-anchored sense primer (NotI_3A_F: GACTGGAAATCCAAAATGTGTGTATCG) and a SacII-anchored antisense primer (SacII_3A_R: GGTGTTTCACTGGGGTCTTTCAAACCA) from mouse genomic DNA and cloned into pMC-DTA-cKO. The targeting vector was linearised with SacII and used for electroporation of the RENKA ES cell lines derived from C57BL/6. Homologous recombination at the *Ubiad1* locus resulted in replacement of the first exon of *Ubiad1* with the neomycin-resistance cassette. Random integration was reduced because of a DTA cassette at the 5′ end of the targeting construct [24]. A total of 5 of 528 neomycin-resistant embryonic stem (ES) clones were correctly targeted (1.67% efficiency), as confirmed by nested PCR and Southern blotting at the 3′-flanking region. Heterozygous ES cell clones were injected into C57BL/6 blastocysts, and two of them formed germline chimaeras that transmitted the targeted allele to their offspring. The resulting male chimeras were mated with C57BL/6 females and their offspring were examined for heterozygosity by Southern blotting and PCR. Heterozygous *Ubiad1* ^+/−^ mice of a C57BL/6 background identified by PCR were viable and fertile. Genotypes were confirmed by PCR with a sense primer (loxP-F, 5′- CCTTGAATTCTCTTCCTGTCGTCGTCTC-3′) and an antisense primer (GTP-R2, 5′-AGTGTTCATAATCCACTGCCAAACC-3′).

### *Ubiad1* ^−/−^ ES cell derivation and ES cell culture

Cryopreserved two-cell embryos obtained by in vitro fertilisation (IVF) were thawed and cultured to blastocyst stage in potassium simplex optimised medium (KSOM medium) at 37°C under 5% CO_2_. ES cell lines were established by a method described previously [25]. In brief, blastocysts were transferred into a 10-cm culture dish with feeder cells and cultured in ES cell medium for 8–10 days at 37°C under 5% CO_2_. When ES cell colonies appeared as cell clumps, each colony was isolated and transferred to a well of a 24-well plate containing feeder cells. The ES cells were subsequently cultured in ES cell medium for 8–10 days. Each ES cell line culture was passaged once before preparing frozen stocks. Genotyping was performed by PCR applied to a DNA template derived from ES cells using the primers described above. ES cells were maintained in Knockout Dulbecco's modified Eagle medium (Gibco BRL) supplemented with 16% knockout serum replacement (KSR; Gibco BRL), 1% non-essential amino acid solution (Invitrogen), 1% glutamine, 0.1 mM beta-mercaptoethanol (Sigma) and LIF solution (ESGRO, 10^7^ units/ml; CHEMICON).

### Morphological analysis of *Ubiad1* ^−/−^ mouse embryos

IVF was performed using unfertilised eggs and sperm prepared from female and male mice carrying a mutation in *Ubiad1*, according to standard methods [26], [27]. Fertilised eggs were cultured to the two-cell embryo stage in KSOM medium at 37°C under 5% CO_2_ [28]. The embryos were cryopreserved by a simple verification method [29]. Besides cryopreservation, some of the embryos were cultured to the blastocyst stage in KSOM at 37°C under 5% CO_2_ to examine their morphology. The cryopreserved two-cell embryos were thawed by the method described previously [28] and washed in KSOM. To evaluate the embryonic development of *Ubiad1* ^−/−^ mice, 10 viable two-cell embryos recovered from cryopreservation were transferred into an oviduct of a pseudopregnant female mouse 1 day after mating with a vasectomised male mouse [30]. The embryos were collected at E7 and E10 to examine their morphology under a dissection microscope. Images of the embryos were captured with a Nikon DXM1200 camera attached to a Nikon TE2000-U microscope for blastocysts and Pixera Pro 600ES with an OLYMPUS SZX9 dissection microscope for E7 and E10 embryos. Genotypes of individual embryos were identified by PCR applied to a DNA template derived from a yolk sac at E10, a whole embryo at E7 or a blastocyst, using forward (loxP-F) and reverse (GTP-R2) primers.

### Mouse embryonic fibroblast isolation

Mouse embryonic fibroblast (MEF) cell cultures were prepared from E15.5 embryos of *Ubiad1* ^+/+^ and *Ubiad1* ^+/−^ mice. The embryos were dissected from the uterus under sterile conditions and washed with PBS. Embryo paws and legs were minced and digested with 0.25% trypsin for 20 minutes at 37°C. Cell suspensions were plated in Dulbecco's modified Eagle medium containing 10% foetal bovine serum and penicillin/streptomycin.

### Measurements of MK-4 and MK-4-d_7_ in tissues of mice administered MD-d_8_

Ten-week-old male *Ubiad1* ^+/+^ and *Ubiad* ^+/−^ mice were orally administered MD-d_8_ as a single dose at 10 µmol/kg body weight. After 24 hours, mice were sacrificed and tissues were removed and stored at −80°C for analysis. MK-4, MK-4 epoxide, MK-4-d_7_ and MK-4-d_7_ epoxide were measured by LC-APCI-MS/MS as reported previously [8], [21].

### Measurements of CoQ9, CoQ10, ^13^C_6_-CoQ9 and ^13^C_6_-CoQ10

Tissues (wet weight, 100–200 mg) were minced and transferred to a brown glass tube with a Teflon-lined screw cap. Next, 0.1 mL of ethanol containing MK-4-^18^O as internal standard, 1.9 mL of ethanol, 1.0 mL distilled water and 3 mL of hexane followed by thorough mixing on a voltex mixer for 5 minutes. The resulting mixture was centrifuged at 2,500 rpm for 5 minutes at 4°C, and the upper layer was transferred to a small brown glass tube and evaporated to dryness under reduced pressure. The residue was dissolved in 1 mL hexane and evaporated under reduced pressure. This residue was dissolved in 60 µL methanol. An aliquot of this solution was analyzed by APCI3000 LC-MS/MS (Applied Biosystems, Foster City, CA). HPLC analyses were performed on a Shimadzu HPLC system (Shimadzu, Kyoto, Japan) consisting of a binary pump (LC-10AD liquid chromatography), an automatic solvent degasser (DGU-14A degasser) and an autosampler (SIL-10AD autoinjector). Separations were performed using a reversed-phase C18 column (Capcell Pak C18 UG120, 5 µm; 4.6 mm inner diameter×250 mm, Shiseido, Tokyo, Japan) with a solvent system consisting of isocratic solvent A. Solvent A contained methanol∶isopropanol (3∶1, v/v) and was delivered at 1.0 mL/minute. This mobile phase was passed through the column at 1.0 mL/minute. The column was maintained at 35°C by a column oven (CTO-10AC column oven). All MS data were collected in positive ion mode with atmospheric pressure chemical ionisation (APCI). The following settings were used: corona discharge needle voltage, 5.5 kV; vaporizer temperature, 400°C; sheath gas (high-purity nitrogen) pressure, 50 p.s.i. and transfer capillary temperature, 220°C. The electron multiplier voltage was set at 850 eV. Identification and quantification were based on MS/MS using multiple reaction monitoring (MRM) mode. The range for the parent scan was 400–900 atomic mass units. MRM transitions (precursor ion and product ion, m/z) and retention time (minute) for each analyte were as follows: MK-4-^18^O: precursor ion, 449.3; product ion, 191.2; retention time, 3.6; CoQ9: precursor ion, 796.5; product ion, 197.1; retention time, 8.7; CoQ10: precursor ion, 864.6; product ion, 197.0; retention time, 11.5; ^13^C_6_-CoQ9: precursor ion, 802.5; product ion, 203.1; retention time, 8.6 and ^13^C_6_-CoQ10: precursor ion, 870.6; product ion, 203.0; retention time, 11.4 [31]–[33]. Calibration, using internal standardisation, was performed by linear regression using five different concentrations of 100, 200, 400, 800 and 1,600 ng/mL.

### Conversion of MD-d_8_ to MK-4-d_7_ and ^13^C_6_-4HB to ^13^C_6_-CoQ9 in ES cells

ES cells described above were maintained in Knockout Dulbecco's modified Eagle medium (Gibco BRL.) supplemented with 16% KSR (Gibco BRL), 1% non-essential amino acid solution (Invitrogen), 1% glutamine, 0.1 mM β-mercaptoethanol (Sigma) and LIF solution (ESGRO, 10^7^ units/ml; CHEMICON). ES cells were cultured on MEF in 6-well tissue culture plates (2×10^5^ cells/well) for 3 days and treated with culture medium containing MD-d_8_ (10^−6^ M) and ^13^C_6_-4HB for 24 hours. ES cells were trypsinised, washed with ES medium and cultured on gelatin-coated plates for 40 minutes. Floated ES cells were collected and washed with cold PBS(−) twice and then stored at −30°C. After warming to room temperature, cells were lysed in 1 mL of PBS(−). Cell lysates (20 µL) were analysed for protein concentrations. PK-^18^O and MK-4-^18^O were added as internal standards to the cell lysates in brown screw-capped tubes. Measurements for MK-4-d_7_, MK-4-d_7_ epoxide and ^13^C_6_-CoQ9 in cells was performed using the method described above.

### Real-time PCR

Total RNA of mouse tissues or ES cells was isolated with Isogen (Nippon Gene) according to the manufacturer's protocol. First-strand cDNA synthesis was performed using AMV reverse transcriptase (TaKaRa). cDNA were mixed with SYBR Green Core Reagent (PE Biosystems), and amplified using the CFX96 real-time PCR system (Bio-Rad). We used mouse *Ubiad1* (GenBank NM_027873, FP:1575-1595, RP:1754-1774) mouse *Coq2* (NM_027978, FP:445-465, RP:555-577), mouse *Gapdh* (GenBank accessions 01289726, FP:633-652, RP:726-745) and mouse *β-actin* (GenBank accessionsX03672, FP:250-271, RP:305-326).

### Western blotting

UBIAD1 expression levels were detected by western blotting. The UBIAD1 antibody was an UBIAD1-specific affinity-purified polyclonal antibody raised in rabbits against an UBIAD1-specific peptide (CPEQDRLPQRSWRQK-COOH) (MRL Co., Ltd.). The peroxidase-conjugated secondary antibody was rabbit Ig raised in donkey (SantaCruz) for 1.5 hours and UBIAD1 protein was detected using an electrochemiluminescent detection system (Nakalai Tesque).

### Administration of MK-4 or CoQ10 to *Ubiad1* ^+/−^ pregnant mice

*Ubiad1* ^+/−^ pregnant mice were orally administered water-soluble CoQ10 (10 µmol/kg/day) or MK-4 (2.25 µmol/kg/day) every other day from 1 day before mating to the day of sacrifice. The doses of oral administration of MK-4 and CoQ10 were decided with reference to previous animal and human studies [34], [35].

### Histology and immunohistochemistry

For histological analysis, embryos were fixed in 4% paraformaldehyde in PBS at 4°C for 20 hours and embedded in a paraffin block. Tissue sections were stained with hematoxylin–eosin (HE). For detection of UBIAD1 protein, we used a monoclonal antibody raised against an amino acid sequence (SKGIDHKKSDDRTLVDRILEPQD) corresponding to the N terminus of the mouse UBIAD1 protein. Formalin-fixed and paraffin-embedded mouse embryonic tissues were deparaffinised and incubated for 30 minutes in 3% hydrogen peroxide/PBS to quench endogenous peroxidases. Sections were rinsed in PBS and immunostained with anti-UBIAD1 antibody at 1∶100 dilution in 0.5% PBS/Ova Albumin at 4°C overnight after antigen retrieval with HistoOne buffer (Nacalai, Kyoto, Japan) for 95°C for 20 minutes. The secondary antibody was HRP-labelled anti-rat IgG antibody (KPL) diluted 1∶1000 in 0.5% PBS/Ova Albumin and incubated for 30 minutes at room temperature. Sections were incubated with Elite ABC Kit (Vector Laboratories) for 30 minutes, rinsed with PBS and detected by staining with DAB (Vector Laboratories) for 2 minutes and counterstaining with methyl green.

### Statistical analysis

Data are expressed as mean ± SEM. Differences between the mean values were analysed using the unpaired Student's *t* test or Dunnett's test: *P<0.05; **P<0.01; ***P<0.001.

## Results

### *Ubiad1*-deficient mice are embryonic lethal

**Figure 1:**
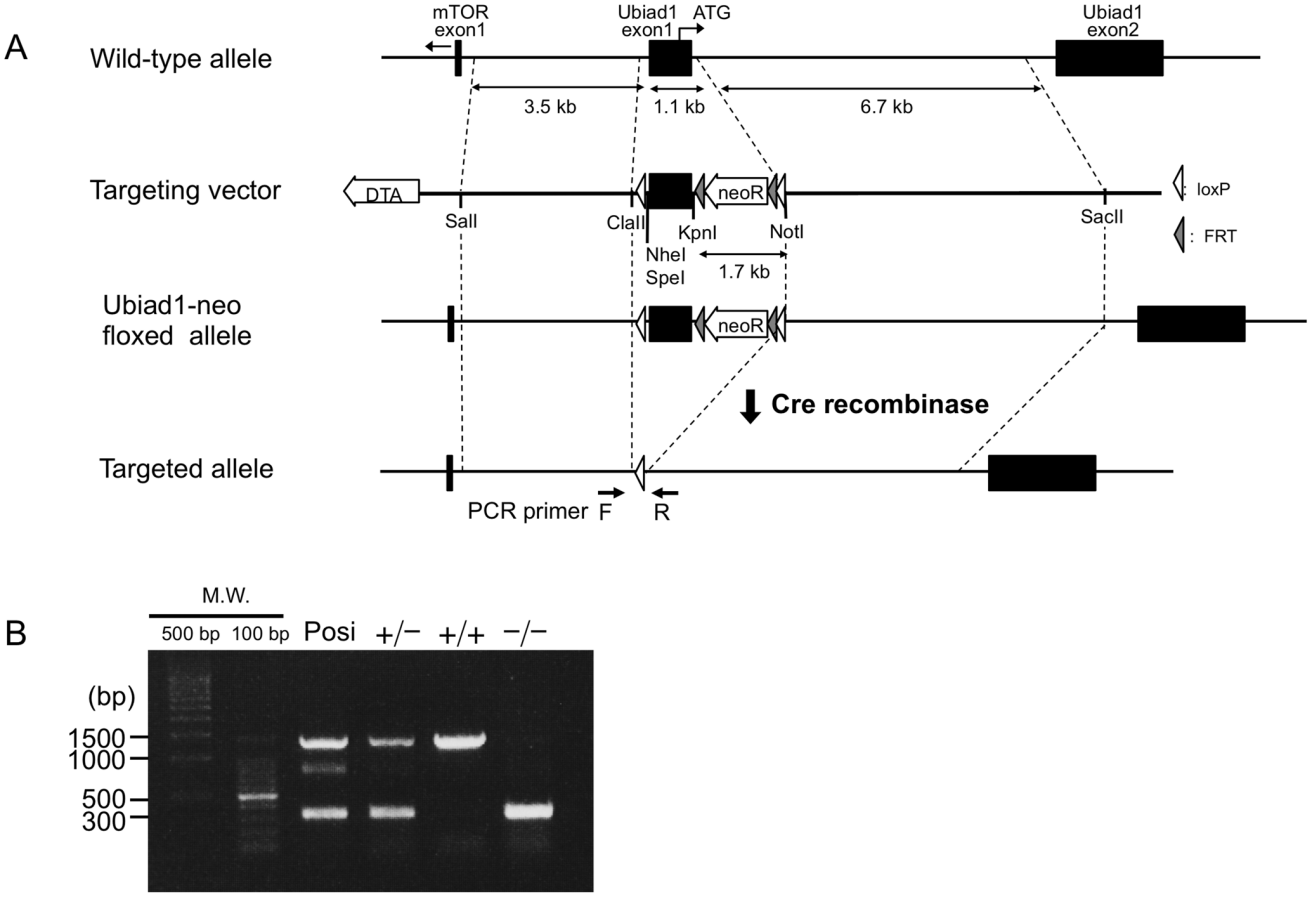
Generation of *Ubiad1* knockout mice. (A) Schematic presentation of ubiad1 genome, targeting vector and disrupted *Ubiad1* genome. (B) PCR genotyping of *Ubiad1* ^+/+^, *Ubiad1* ^+/−^ and *Ubiad1* ^−/−^ embryos. PCR genotyping of tail DNA of *Ubiad1* ^+/+^, *Ubiad1* ^+/−^ and *Ubiad1* ^−/−^ embryos. Lane 1, positive controls for *Ubiad1* ^+/−^ allele. Lane 2, PCR bands of *Ubiad1* ^+/−^ embryos. Lane 3, PCR bands of *Ubiad1* ^+/+^ embryos. Lane 4, PCR bands of *Ubiad1* ^−/−^ embryos.

**Figure 2:**
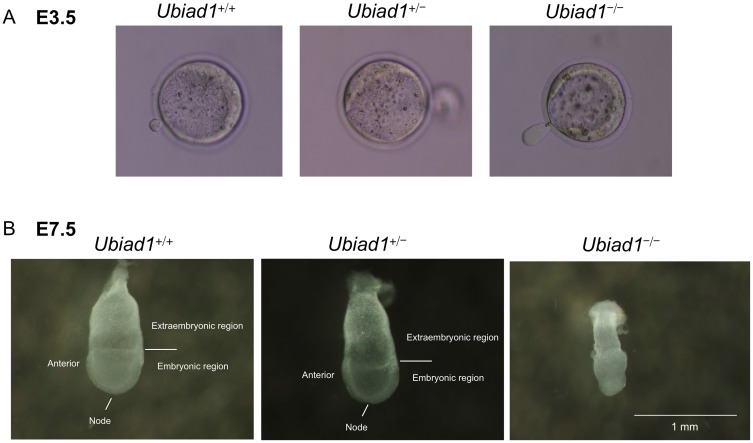
Developmental arrest in *Ubiad1* knockout embryos. (A) Morphology of E3.5 blastocysts. Blastocysts were cultured from in vitro fertilised embryos of *Ubiad1* ^+/+^, *Ubiad1* ^+/−^ and *Ubiad1* ^−/−^ mice. (B) Morphology of E7.5 embryos.

Mice *Ubiad1* contains two exons. To disrupt *Ubiad1*, the targeting vector was designed to flank exon 1 with two *loxP* sequences, and a frameshift was generated by excision with Cre recombinase (Figure 1). *Ubiad1* ^+/−^ mice having a C57BL/6 background identified by PCR were viable and fertile. In comparison to control littermates (*Ubiad1* ^+/+^), they did not exhibit any overt phenotype and had similar body size and weight (Figure S1). Male and female *Ubiad1* ^+/−^ mice were intercrossed to obtain *Ubiad1* ^−/−^ mice. However, no *Ubiad1* ^−/−^ pup could be identified among over 150 analysed offspring, suggesting that the disruption of *Ubiad1* leads to embryonic lethality. In contrast, *Ubiad1* ^+/+^ and *Ubiad1* ^+/−^ mice were obtained at the Mendelian frequency (Table 1). To assess the time of death in utero, IVF were performed using sperm and eggs obtained from male and female *Ubiad1* ^+/−^ mice, respectively and the embryos in utero of pseudopregnant *Ubiad1* ^+/+^ mice were dissected and genotyped at several gestation times from E3.5 to E10.5. At E3.5, *Ubiad1* ^−/−^ blastocysts were microscopically indistinguishable from *Ubiad1* ^+/+^ and *Ubiad1* ^+/−^ blastocysts (Figure 2A). However, at E7.5, both *Ubiad1* ^+/+^ and *Ubiad1* ^+/−^ embryos showed elongation of the egg cylinder, which developed to a primitive streak with the formation of mesoderm between ectoderm and endoderm. In contrast, *Ubiad1* ^−/−^ embryos were consistently smaller than their littermates and exhibited no signs of primitive streak formation. The constriction marking the extraembryonic-embryonic ectoderm border was absent, and mesoderm had not formed in *Ubiad1* ^−/−^ embryos (Figure 2B). At E10.5, *Ubiad1* ^−/−^ embryos were not found at all, similar to the results observed in the intercrosses of male and female *Ubiad1* ^+/−^ mice in vivo.

**Table 1:** Analysis of embryos and weaning from intercrosses of *Ubiad1* ^+/−^ mice.

Stage	Total offspring	*Ubiad1* ^+/+^	*Ubiad1* ^+/−^	*Ubiad1* ^−/−^	Resorbed
E3.5	19	3	13	3	0
E7.5	56	7	22	13	14
E10.5	103	15	51	0	37
E15.5	111	20	69	0	22
Weanling	151	49	102	0	

### *Ubiad1* ^−/−^ ES cells were unable to synthesize MK-4, but able to synthesize CoQ9 similar to wild-type ES cells

**Figure 3:**
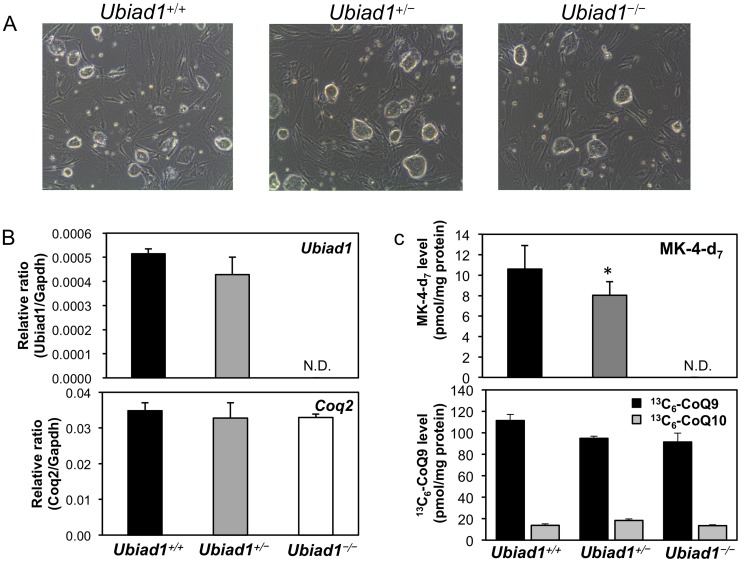
*Ubiad1* and *Coq2* expression and the biosynthesis of MK-4 and CoQ9 in ES cells derived from *Ubiad1* ^+/+^, *Ubiad1* ^+/−^ and *Ubiad1* ^−/−^ embryos. (A) Morphology of *Ubiad1* ^+/+^, *Ubiad1* ^+/−^ and *Ubiad1* ^−/−^ ES cells. (B) *Ubiad1* and *Coq2* mRNA expression in *Ubiad1* ^+/+^, *Ubiad1* ^+/−^ and *Ubiad1* ^−/−^ ES cells. (C) The biosynthesis of MK-4-d_7_, ^13^C_6_-CoQ9 and ^13^C_6_-CoQ10 in *Ubiad1* ^+/+^, *Ubiad1* ^+/−^ and *Ubiad1* ^−/−^ ES cells. Mean ± s.e.m. Dunnett's test, *P<0.05. N.D.: not detected.

*Ubiad1* ^−/−^ mice are embryonic lethal. For alternative confirmation of the successful ablation of *Ubiad1*, we established ES cells from fertilised ova of *Ubiad1* ^+/−^ mice. *Ubiad1* ^−/−^ ES cells exhibited neither *Ubiad1* mRNA and protein expression nor MK-4 synthesis activity, whereas they exhibited CoQ9 or CoQ10 synthesis activity and *coenzyme Q2 4-hydroxybenzoate-polyprenyltransferase* (*Coq2*) mRNA expression similar to those of *Ubiad1* ^+/+^ and *Ubiad1* ^+/−^ ES cells (Figure 3). These data exclude the presence of an additional biosynthesis pathway of MK-4 and indicate that UBIAD1 is the sole MK-4 biosynthetic enzyme in embryonic development of mice.

### Neither MK-4 nor CoQ10 treatment to pregnant *Ubiad1* ^+/−^ mice rescued their *Ubiad1* ^−/−^ embryos from lethality, but extended the lifespan of many *Ubiad1* ^−/−^ embryos to term

**Figure 4:**
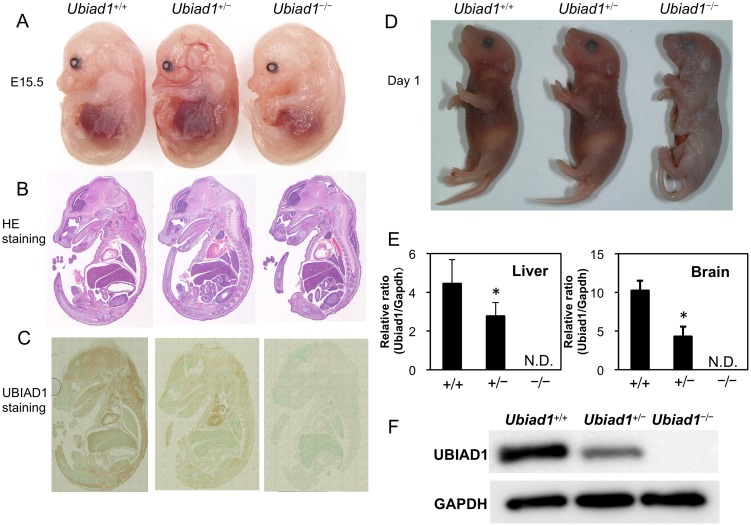
Morphological examination of *Ubiad1* ^+/+^, *Ubiad1* ^+/−^ and *Ubiad1* ^−/−^ embryos and weanling mice (postnatal day 1) from pregnant *Ubiad1* ^+/−^ mice orally administered CoQ10. (A) Morphology of *Ubiad1* ^+/+^, *Ubiad1* ^+/−^ and *Ubiad1* ^−/−^ embryos at E15.5 from CoQ10-supplemented pregnant *Ubiad1* ^+/−^ mice. (B) HE staining of embryos at E15.5. (C) Immunohistochemical staining of UBIAD1 in *Ubiad1* ^+/+^, *Ubiad1* ^+/−^ and *Ubiad1* ^−/−^ embryos at E15.5. (D) Morphology of *Ubiad1* ^+/+^, *Ubiad1* ^+/−^ and *Ubiad1* ^−/−^ embryos at postnatal day 1. *Ubiad1* ^+/+^ and *Ubiad1* ^+/−^ mice were born alive, but *Ubiad1* ^−/−^ mice were stillborn. (E) *Ubiad1* mRNA expression in the livers and brains of *Ubiad1* ^+/+^, *Ubiad1* ^+/−^ and *Ubiad1* ^−/−^ embryos at E15.5 from CoQ10-supplemented pregnant *Ubiad1* ^+/−^ mice. Mean ± s.e.m. Dunnett's test, *P<0.05. N.D.: not detected. (F) UBIAD1 expression in the brains of *Ubiad1* ^+/+^, *Ubiad1* ^+/−^ and *Ubiad1* ^−/−^ embryos at E15.5 from CoQ10-supplemented pregnant *Ubiad1* ^+/−^ mice.

**Figure 5:**
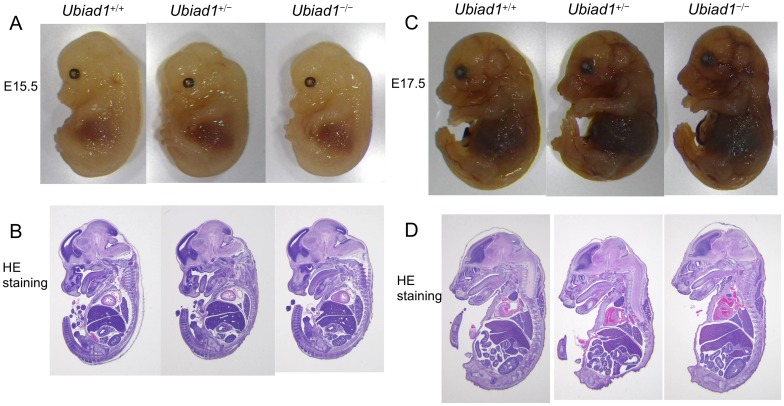
Morphological examination of *Ubiad1* ^+/+^, *Ubiad1* ^+/−^ and *Ubiad1* ^−/−^ embryos from pregnant *Ubiad1* ^+/−^ mice orally administered MK-4. (A) Morphology of *Ubiad1* ^+/+^, *Ubiad1* ^+/−^ and *Ubiad1* ^−/−^ embryos at E15.5 from MK-4-supplemented pregnant *Ubiad1* ^+/−^ mice. (B) HE staining of embryos at E15.5. (C) Morphology of *Ubiad1* ^+/+^, *Ubiad1* ^+/−^ and *Ubiad1* ^−/−^ embryos at E17.5 from MK-4-supplemented pregnant *Ubiad1* ^+/−^ mice. (D) HE staining of embryos at E17.5.

To examine whether MK-4 or CoQ10 treatment rescues the embryonic lethality of *Ubiad1* ^−/−^ mice, either MK-4 or CoQ10 at a dose of 10 µmol/kg/day or 2.25 µmol/kg/day was orally administered to *Ubiad1* ^+/−^ pregnant mice from 1 day before crossing and throughout pregnancy. As a result, approximately 1.8% of *Ubiad1* ^−/−^ embryos survived by E15.5 and approximately 12.5% of *Ubiad1* ^−/−^ embryos survived by day 1 in MK-4-treated *Ubiad1* ^+/−^ pregnant mice. In CoQ10-treated *Ubiad1* ^+/−^ pregnant mice, approximately 2.2% of *Ubiad1* ^−/−^ embryos survived by E15.5 and approximately 1.8% of *Ubiad1* ^−/−^ embryos survived by day 1, although no *Ubiad1* ^−/−^ embryo was identified at E15.5 in the non-treated *Ubiad1* ^+/−^ pregnant mice (Table 2). A *Ubiad1* ^−/−^ embryo at E15.5 from the *Ubiad1* ^+/−^ mice orally administered CoQ10 throughout pregnancy were indistinguishable from their *Ubiad1* ^+/+^ and *Ubiad1* ^+/−^ littermates (Figure 4A–C). A weanling *Ubiad1* ^−/−^ mouse on day 1 from the *Ubiad1* ^+/−^ mice orally administered CoQ10 throughout pregnancy died immediately after birth or stillbirth (Figure 4D). The cause of death was not identified. In this *Ubiad1* ^−/−^ mouse, we could find no corneal and hemorrhagic abnormalities that had been observed in SCD patients [15] or *ubiad1*-mutated zebrafish [12], [14]. The size and morphological features of *Ubiad1* ^−/−^ embryo at E15.5 and E17.5 from the *Ubiad1* ^+/−^ mice orally administered MK-4 throughout pregnancy did not differ from those of their *Ubiad1* ^+/+^ and *Ubiad1* ^+/−^ littermates (Figure 5A–D). A weanling *Ubiad1* ^−/−^ mouse on day 1 was not alive. UBIAD1 mRNA and protein expression in *Ubiad1* ^−/−^ embryos at E15.5 was completely abolished by the targeting, as demonstrated by real-time RT-PCR and western blotting (Figure 4E, F). These results suggest that MK-4 or CoQ10 treatment would help to rescue the embryonic lethality of *Ubiad1* ^−/−^ mice, although their contribution might be small and limited.

**Table 2:** Analysis of embryos and weaning from intercrosses of *Ubiad1* ^+/−^ mice rescued by MK-4 or CoQ10.

Stage	Total offspring	*Ubiad1* ^+/+^	*Ubiad1* ^+/−^	*Ubiad1* ^−/−^	Resorbed
Rescured by MK-4					
E15.5	113	25	70	2	16
Day 1	24	6	15	3 leathal	
Rescured by CoQ10					
E15.5	270	71	147	6	46
Day 1	147	43	101	3 leathal	

### Levels of MK-4 and its epoxide in tissues of *Ubiad1* ^+/−^ mice are approximately half of those in tissues of *Ubiad1* ^+/+^ mice at 28 weeks of age

PK originating in a laboratory chow diet was found in all (*n* = 23) tissues measured, although at widely varying levels, whereas PK epoxide was found in only five and six tissues of *Ubiad1* ^+/+^ and *Ubiad1* ^+/−^ mice, respectively. There was no significant difference between both groups in the levels of PK and its epoxide. In contrast, MK-4 was found in all tissues measured, although at widely varying levels. MK-4 epoxide was found in 17 and 12 tissues of *Ubiad1* ^+/+^ and *Ubiad1* ^+/−^ mice, respectively. Again, levels of MK-4 in the tissues of *Ubiad1* ^+/−^ mice were approximately half of those in tissues of the wild type (Table S1).

### Levels of CoQ9 and CoQ10 in tissues of *Ubiad1* ^+/−^ mice are similar to those of *Ubiad1* ^+/+^ mice at 28 weeks of age

In *Ubiad1* ^+/+^ and *Ubiad1* ^+/−^ mice, both CoQ9 and CoQ10 were found in all tissues measured, although at widely varying levels; there was no significant difference in levels between the two groups (Table S2). We first measured the concentrations of MK-4, MK-4 epoxide, CoQ9 and CoQ10 in five tissues (cerebrum, heart, liver, kidney and small intestine) that are known to be important and representative tissues of vitamin K and CoQ functions. As a result, the concentrations of MK-4 and MK-4 epoxide in the above tissues of *Ubiad1* ^+/−^ mice were found likely to be approximately half of those of *Ubiad1* ^+/+^ mice (Table S1), whereas the concentrations of CoQ9 and CoQ10 in the same tissues of *Ubiad1* ^+/−^ mice were found likely to be similar to those of *Ubiad1* ^+/+^ mice (Table S2). To confirm the influence of *Ubiad1* ablation, we further measured the concentrations of MK-4 and MK-4 epoxide in additional 18 tissues of both *Ubiad1* ^+/−^ and *Ubiad1* ^+/+^ mice (Table S1). It is obvious that UBIAD1 is the enzyme responsible for the synthesis of MK-4, but not CoQ9 and CoQ10 in mice.

### Serum levels of total cholesterol, free cholesterol and HDL-cholesterol in *Ubiad1* ^+/−^ mice are higher than those in *Ubiad1* ^+/+^ mice at 28 weeks of age

In blood chemical analysis, values of total cholesterol, free cholesterol and HDL-cholesterol in *Ubiad1* ^+/−^ mice were significantly higher than those in *Ubiad1* ^+/+^ mice. However, values of calcium, phosphorus, glucose, LDL-cholesterol and triglyceride were not significantly different between the two groups (Table S3). These results suggest that UBIAD1 deficiency may affect cholesterol metabolism in mice similarly in human SCD patients [15], [36].

### UBIAD1 expression and MK-4 synthesis activity in the cerebrum of *Ubiad1* ^+/−^ mice are at approximately half of wild-type levels at 28 weeks old

UBIAD1 mRNA and protein expression levels were evaluated by real-time RT-PCR and Western blot analysis, respectively. As expected, significant reductions in UBIAD1 mRNA and protein were observed compared to wild-type levels. Similar reductions in MK-4 biosynthetic activity in the cerebrum of the *Ubiad1* ^+/−^ mice were observed compared to the wild-type mice (Figure S2).

### *Ubiad1* ^+/−^ MEF cells exhibit approximately half the MK-4 synthetic activity of the *Ubiad1* ^+/+^ MEF cells, but CoQ9 synthetic activity is similar between both genotypes of MEF cells

As *Ubiad1* ^−/−^ embryos uniformly die beyond E7.5, we generated MEF cells from *Ubiad1* ^+/+^ and *Ubiad1* ^+/−^ embryos at E15.5 and measured their MK-4 and CoQ9 synthetic activities. Both *Ubiad1* ^+/+^ and *Ubiad1* ^+/−^ MEF cells grew normally and were indistinguishable from each other microscopically. Similarly to the results for the ES cells, *Ubiad1* ^+/−^ MEF cells exhibited approximately half of the MK-4 synthetic activity of *Ubiad1* ^+/+^ MEF cells, whereas both genotypes of MEF cells exhibited similar CoQ9 synthetic activity, suggesting that UBIAD1 is a MK-4 synthetic enzyme, but may not be a CoQ9 synthetic enzyme in mice. (Figure S3).

## Discussion

UBIAD1 is a recently identified MK-4 biosynthetic enzyme in mice and humans. UBIAD1 is expressed and coexists with vitamin K throughout the body, suggesting various physiological functions of vitamin K. We showed, for the first time to our knowledge, that *Ubiad1* knockout mice uniformly failed to survive beyond E7.5, exhibiting a small-sized body and prominent gastrulation arrest. Oral administration of MK-4 or CoQ10 to *Ubiad1* ^+/−^ pregnant mice crossed with male *Ubiad1* ^+/−^ mice resulted in the appearance of a few *Ubiad1* ^−/−^ embryos from E10.5 to term, but no pup was observed alive after birth. These results suggest that both MK-4 and CoQ10 can only partially rescue the *Ubiad1* ^−/−^ embryonic lethal phenotype. Hitherto, there have been inconsistent reports with respect to the functions of *Ubiad1* as a MK-4 and/or CoQ10 biosynthetic enzyme. Hegarty et al. reported that *ubiad1*-generated MK-4 rescued the zebrafish *ubiad1* vascular integrity/maintenance mutant *reddish^S587^* (*reh*), which develops a functional vasculature by 24 to 36 hours after fertilisation, but then displays cranial vascular haemorrhages because of vascular degeneration by 48 hours after fertilisation [14]. In contrast, CoQ10 was unable to rescue the *reh* vascular phenotype. Vos et al. reported that *Drosophila ubiad1/heix* is a modifier of *pink 1*, a gene mutated in Parkinson's disease with a defect of mitochondrial function, and that MK-4 but not CoQ10 rescued the *ubiad1/heix* mutant phenotype [7]. In contrast, Mugoni et al. recently reported that UBIAD1 is a non-mitochondrial CoQ10 synthetic enzyme with specific cardiovascular protective function via modulation of eNOS activity, and that loss of UBIAD1 induces cardiovascular failure in zebrafish embryos by increasing oxidative stress [12]. Though it remains uncertain whether UBIAD1 in zebrafish and Drosophila is able to synthesize MK-4 like humans and mice [10], it is obvious that mutations in *ubiad1* lead to severe or lethal cardiovascular failure in these species. Considering these findings, complete loss of *Ubiad1* function as observed in the present study may lead to a cardiovascular defect in a mouse embryo, leading in turn to foetal demise. To further elucidate the function of UBIAD1, it will be necessary to analyse the *Ubiad1* knockout mouse phenotype, but such an analysis is currently made difficult by the uniform death of *Ubiad1* knockout mice beyond E7.5 and the very low numbers able to survive from mid-embryonic stage to term with supplementation with MK-4 or CoQ10. To overcome this limitation, we are currently generating tissue-specific *Ubiad1* knockout mice that will develop normally and will enable us to determine whether UBIAD1 regulates vascular integrity/maintenance in mice, as observed in zebrafish and *Drosophila*.

**Figure 6:**
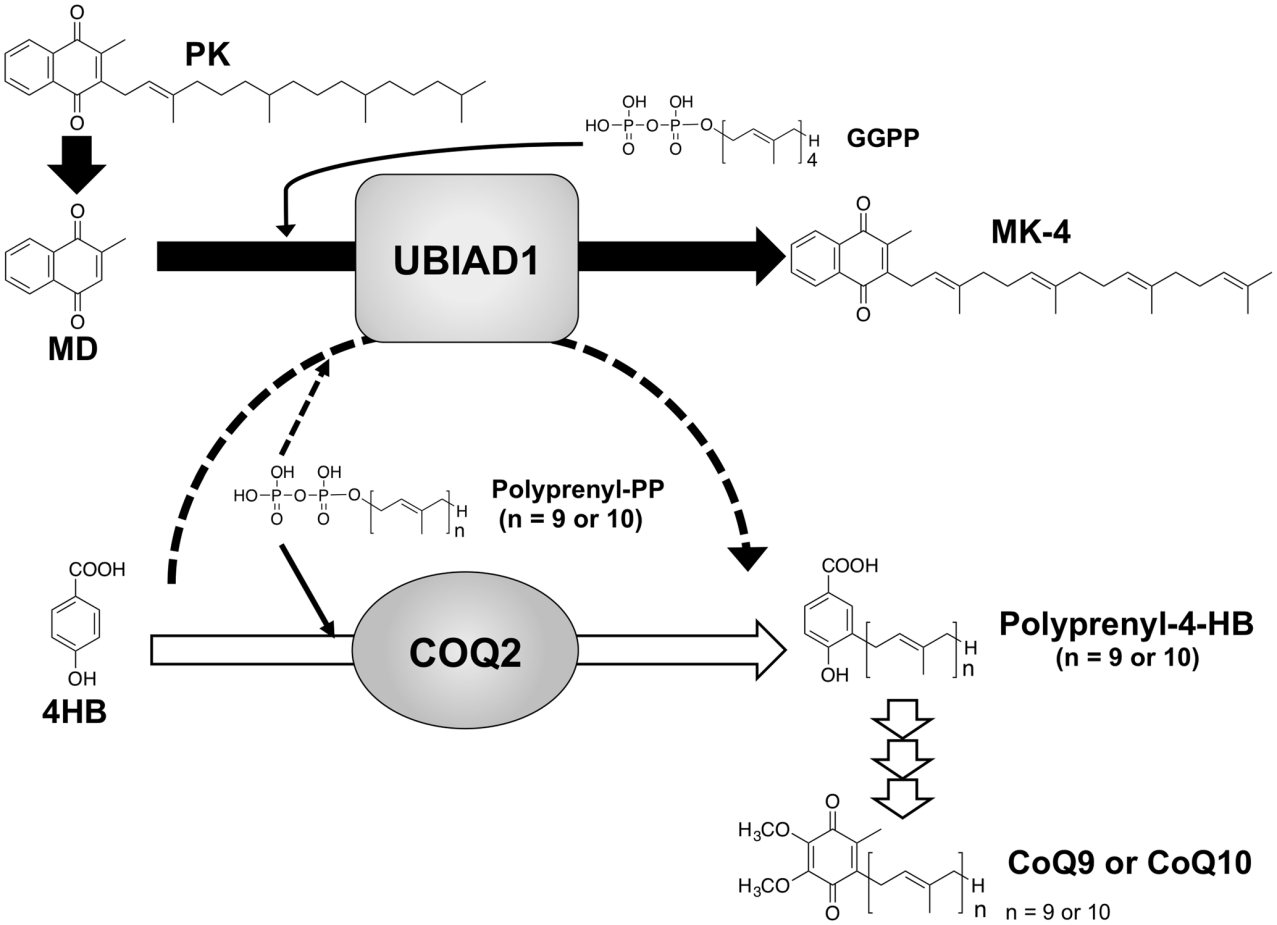
MK-4 and CoQ9/CoQ10 biosynthetic mechanisms of UBIAD1 and COQ2 in mammals. MD is released from PK in the intestine and converted to MK-4. ^13^C_6_4-HB is prenylated to polyprenyl-4-HB by COQ2. Polyprenyl-4-HB is finally converted to CoQ9/CoQ10 by several enzymes.

It remains unclear whether UBIAD1 can synthesize MK-4 and/or CoQ9 in mice. Mugoni et al. reported that zebrafish can synthesize CoQ10 but not MK-4 [12]. However, we previously reported that short interfering RNA treatment against the UBIAD1 gene and the transfection of UBIAD1 expression vector in human osteoblast-like MG-63 cells resulted in a marked reduction and a significant increase of the biosynthesis of MK-4, respectively. We further confirmed that microsomes prepared from human UBIAD1 baculovirus-infected Sf9 cells catalyse the biosynthesis of MK-4 in a dose-dependent manner [10]. In the present study, *Ubiad1* ^−/−^ ES cells exhibited neither UBIAD1 mRNA and protein expression nor biosynthesis activity of MK-4 (Figure 3) and *Ubiad1* ^+/−^ ES cells exhibited approximately half of the MK-4 synthetic activity of *Ubiad1* ^+/+^ ES cells. *Ubiad1* ^+/−^ MEF cells exhibited approximately half of the MK-4 synthetic activity of *Ubiad1* ^+/+^ MEF cells; however, CoQ9 synthetic activity was similar among the three genotypes of ES cells and also between both genotypes of MEF cells. Levels of tissue MK-4 and MK-4 synthesis activity of the cerebrum of *Ubiad1* ^+/−^ mice were approximately half of those of *Ubiad1* ^+/+^ mice at 28 weeks old (Table S1). These findings may exclude the existence of an MK-4 biosynthetic enzyme other than UBIAD1, and suggest that UBIAD1 is the sole MK-4 biosynthetic enzyme at least in embryonic development of mice. However, it is uncertain whether UBIAD1 is a CoQ9 biosynthetic enzyme in mice, given that we could observe no significant difference in concentrations of CoQ9 between the tissues of *Ubiad1* ^+/+^ and *Ubiad1* ^+/−^ mice, and that *Ubiad1* ^−/−^ ES cells exhibited CoQ9 synthesis activity similar to that of *Ubiad1* ^+/+^ ES cells. At present, we have no explanation for the inconsistency of our results with those reported by Mugoni et al. [12]. One possible explanation may be the difference in the production rate of CoQ9 in the mitochondrial and Golgi membrane compartments. CoQ9 is well known to be generated predominantly by a mitochondrial enzyme COQ2 that catalyses the conversion of ^13^C_6_-4HB to 3-solanesyl-4HB, the first and rate-limiting step in the biosynthesis pathway of CoQ9 (Figure 6). Mugoni et al. reported that UBIAD1 is a non-mitochondrial CoQ9 biosynthetic enzyme in the Golgi membrane compartment [12]. It is thus plausible that the amounts of CoQ9 generated by UBIAD1 in the Golgi membrane are too small, compared to the amounts generated by COQ2 in the mitochondria, to show significant differences at the tissue and cellular CoQ9 levels. To date there are several conflicting reports showing higher or lower concentrations of CoQ9 in the mitochondrial membrane compartment than in the Golgi membrane compartment. When we measured separately the concentrations of CoQ9 in the mitochondria and Golgi membrane compartments of *Ubiad1* ^+/−^ and *Ubiad1* ^+/+^ mice, we observed no marked difference in the concentrations of CoQ9 between both genotypes of mice (data not shown). To determine whether UBIAD1 is responsible for the biosynthesis of CoQ9, it would be more effective to determine whether the microsomes prepared from mouse UBIAD1 baculovirus-infected Sf9 cells catalyse the conversion of ^13^C_6_-4HB to 3-solanesyl-4HB in vitro. However, an authentic preparation of 3-solanesyl-4HB, required for the evaluation of the enzyme reaction, is not currently commercially available, and chemical synthesis of this compound is currently being undertaken in our laboratory.

In the present study, oral supplementation with MK-4 or CoQ10 to pregnant *Ubiad1* ^+/−^ mice only partially rescued their *Ubiad1* ^−/−^ embryos *in utero*, but failed to rescue them by term, raising the possibility that UBIAD1 has additional roles beyond its role in synthesizing MK-4 and/or CoQ9. Hegarty et al. reported that mutations in *ubiad1* cause severe vascular defects and cardiac defects in zebrafish and that endothelial/endocardial expression of wild-type *ubiad1* in the mutants led to rescue of both vascular and cardiac functions; however, MK-4 treatment rescued vascular but not cardiac phenotype. Warfarin-treated zebrafish exhibited atretic cranial vasculature and haemorrhaging, owing to endothelial cell apoptosis [14]. Interestingly, these warfarin-treated zebrafish did not develop appreciable cardiac oedema, supporting the idea that UBIAD1 possesses additional functions besides the biosynthesis MK-4 that regulate endocardial and myocardial functions. In human studies, UBIAD1 has been implicated in SCD, a rare autosomal-dominant disease associated with at least one of 22 different heterozygous *UBIAD1* missense mutations. Surprisingly, mutations in *UBIAD1* in SCD patients are not necessarily associated with their MK-4 synthetic activity, given that we observed that some mutations have weak but others strong activity [36]. In this study, serum concentrations of total cholesterol, free cholesterol and HDL-cholesterol in *Ubiad1* ^+/−^ mice were significantly higher than those of *Ubiad1* ^+/+^ mice (Table S3). SCD has been reported to be characterized by the abnormal deposition of cholesterol, phospholipids and HDL apolipoproteins in the corneas [37]. Although there is a difference between cornea in SCD patients and blood in *Ubiad1* ^+/−^ mice, it is common that values of total cholesterol and HDL-cholesterol are consistently high on accounts of UBIAD1 mutation and deletion. Consequently, these results suggest that UBIAD1 may affect cholesterol metabolism in mice similarly in human SCD patients [15], [36].

UBIAD1 (also known as TERE1) message and protein expression is reduced in human bladder transitional cell carcinoma (TCC) and metastatic prostate cancer. TERE1/UBIAD1 overexpression inhibits the growth of TCC cell lines and prostate cancer cell lines, although no *UBIAD1* mutations have been identified in individuals with TCC, suggesting that UBIAD1 synthesizes anti-tumour proliferating metabolites other than MK-4 or interacts directly with anti-cancer modulators [17]–[20]. To date, no homozygous *UBIAD1* mutations have been reported in animals or humans. Considering our present results in mice, complete loss of UBIAD1 function may lead to severe phenotypes, such as severe vascular and cardiac defects, which may lead to embryonic lethality. We are uncertain whether phenotypes observed in *Ubiad1* ^−/−^ mice can be linked to SCD family genetics because they are embryonic lethal. It would be interesting to investigate any abnormalities in corneas in corneal-specific *Ubiad1* ^−/−^ mice, if they are alive after birth. Nickerson et al. have recently reported complex molecular links between enzymes such as UBIAD1 and HMGCR or SOAT1 catalysing vitamin K and cholesterol metabolism, respectively, and an interaction between UBIAD1 and the cholesterol transport protein, apoE [36]. Because UBIAD1 is considered to act in a complex with HMGCR, SOAT1 or ApoE on cholesterol metabolism [36], the phenotype of *Ubiad1* ^−/−^ mice may serve to explain the role of multi-protein complexes in cholesterol metabolism and SCD genetics. Thus, future studies investigating additional functions of UBIAD1 beyond the canonical functions of vitamin K are warranted, with the aim of elucidating the physiological and pathophysiological roles of UBIAD1 in cardiovascular homeostasis.

There were several limitations to this study. First, the number of *Ubiad1* ^−/−^ embryos observed beyond E10.5, even supplemented with supra-physiological doses of MK-4 or CoQ10 were quite few and no pup was observed alive after birth. For this reason we were unable to analyse in detail the *Ubiad1* ^−/−^ embryo phenotype morphologically and histologically. In the present study, the *Ubiad1* ^−/−^ embryo was partially rescued by not only MK-4 but also CoQ10, although UBIAD1 may not be a likely CoQ9 and/or CoQ10 synthetic enzyme. We are uncertain why the phenotype of *Ubiad1* ^−/−^ embryo was alleviated by CoQ10 treatment. Hegarty et al. recently reported that the phenotype of *ubiad1*-mutant zebrafish was rescued by MK-4 but not CoQ10 treatment [14]. In contrast, Mugoni et al. reported that the phenotype of *ubiad1*-mutant zebrafish was rescued by CoQ10 but not MK-4 treatment [12]. Given that both MK-4 and CoQ10 have an anti-oxidative effect and function as electron carriers in cells, CoQ10 treatment may have served partially to compensate the roles of anti-oxidation and electron transport of MK-4, which was completely abolished in *Ubiad1* ^−/−^ mice. Second, we were unable to obtain in vivo evidence as to whether UBIAD1 is a CoQ9 synthetic enzyme in mice. To confirm this possibility, it would be more effective to examine. whether microsomes prepared from UBIAD1 baculovirus-infected Sf9 cells catalyse the conversion of ^13^C_6_-4HB to 3-solanesyl-4HB, the first product in the biosynthesis pathway of CoQ9. However, 3-solanesyl-4HB is not commercially available, and chemical synthesis of this compound is currently being undertaken in our laboratory.

In summary, the present study shows for the first time that UBIAD1 is the sole enzyme responsible for the biosynthesis of MK-4 in the tissues of mice and the complete ablation of the *Ubiad1* gene leads to embryonic lethality. Rescued only partially by oral supplementation with MK-4 or CoQ10, UBIAD1 may play a critical role in embryonic development through the biosynthesis of MK-4, but an alternative UBIAD1/vitamin K-independent pathway may be involved in the embryonic development of mice. Given that systemic *Ubiad1* knockout mice uniformly die between E7.5 to E10.5, the elucidation of the physiological and pathophysiological roles of *Ubiad1* will require the generation of mice exhibiting tissue-specific deficiency of *Ubiad1* without embryonic and postnatal lethality.

## Supporting Information

Figure S1 **Body weight curves of male and female** ***Ubiad1*** **^+/+^ and heterozygous** ***Ubiad1*** **^+/−^ mice.** (A) Body weight curves of male *Ubiad1* ^+/+^ and *Ubiad1* ^+/−^ mice (n = 10/genotype). (B) Body weight curves of female *Ubiad1* ^+/+^ and *Ubiad1* ^+/−^ mice (n = 10/genotype). Mean ± s.e.m.
(TIF)

Figure S2 ***Ubiad1*** **expression and MK-4 synthesis activity in the cerebrum of** ***Ubiad1*** **^+/+^ and** ***Ubiad1*** **^+/−^ mice.** (A) *Ubiad1* mRNA expression in the cerebrum of *Ubiad1* ^+/+^ and *Ubiad1* ^+/−^ mice (28 weeks old). (B) UBIAD1 protein expression in the cerebrum of *Ubiad1* ^+/+^ and *Ubiad1* ^+/−^ mice (28 weeks old). (C) The biosynthesis of MK-4-d_7_ from MD-d_8_ in the cerebrum of *Ubiad1* ^+/+^ and *Ubiad1* ^+/−^ mice (10 weeks old). Mean ± s.e.m. Student's *t* test, *P<0.05 and **P<0.01. N.D.: not detected.
(TIF)

Figure S3 **The biosynthesis of MK-4 from MD-d_8_ and that of CoQ9/CoQ10 from ^13^C_6_-4HB in MEF from** ***Ubiad1*** **^+/+^ and** ***Ubiad1*** **^+/−^ mice embryo.** (A) Morphology of *Ubiad1* ^+/+^ and *Ubiad1* ^+/−^ MEF cells. (B) The biosynthesis of MK-4-d_7_ and its epoxide from MD-d_8_ in MEF cells. (C) The biosynthesis of ^13^C_6_-CoQ9 and ^13^C_6_-CoQ10 from ^13^C_6_-4HB in MEF cells. Mean ± s.e.m. N.D.: not detected. * P<0.05.
(TIF)

Table S1 **Concentrations of PK, MK-4 and their epoxides in the tissues of** ***Ubiad1*** **^+/+^ and** ***Ubiad1*** **^+/−^ mice (28 weeks old).**
(DOCX)

Table S2 **Concentrations of CoQ9 and CoQ10 in the tissues of** ***Ubiad1*** **^+/+^ and** ***Ubiad1*** **^+/−^ mice (28 weeks old).**
(DOCX)

Table S3 **Blood chemical values of** ***Ubiad1*** **^+/+^ and** ***Ubiad1*** **^+/−^ mice (28 weeks old).**
(DOCX)
