# Supplementary figures and images for: Vitamin K2 Biosynthetic Enzyme, UBIAD1 Is Essential for Embryonic Development of Mice
Source: PLoS One. 2014 Aug 15;9(8):e104078. doi: 10.1371/journal.pone.0104078 (PMC4134213; doi:10.1371/journal.pone.0104078)

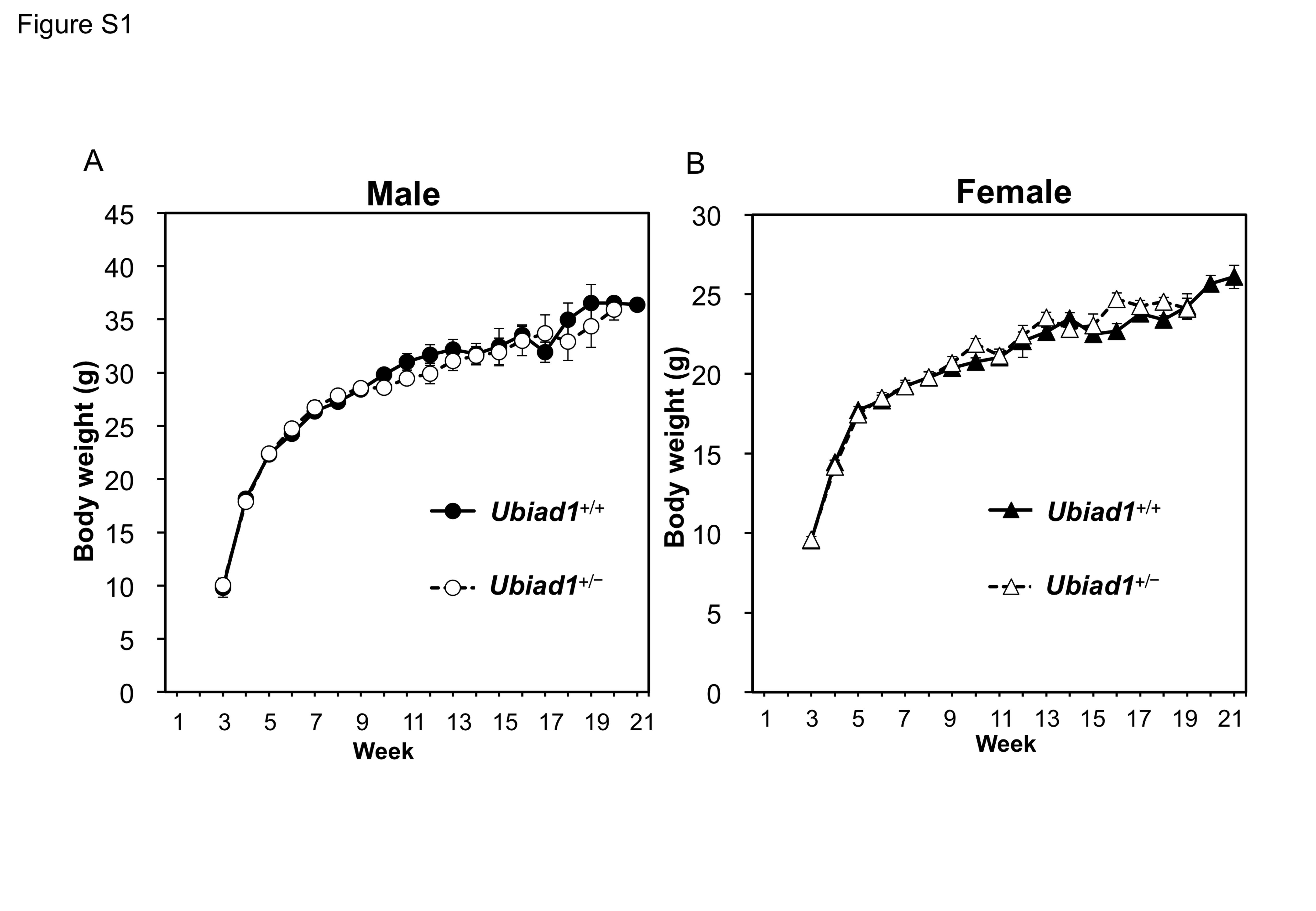

Supplement: Figure S1 — Body weight curves of male and female Ubiad1 +/+ and heterozygous Ubiad1 +/− mice. (A) Body weight curves of male Ubiad1 +/+ and Ubiad1 +/− mice (n = 10/genotype). (B) Body weight curves of female Ubiad1 +/+ and Ubiad1 +/− mice (n = 10/genotype). Mean ± s.e.m. (TIF) [file pone.0104078.s001.tif]

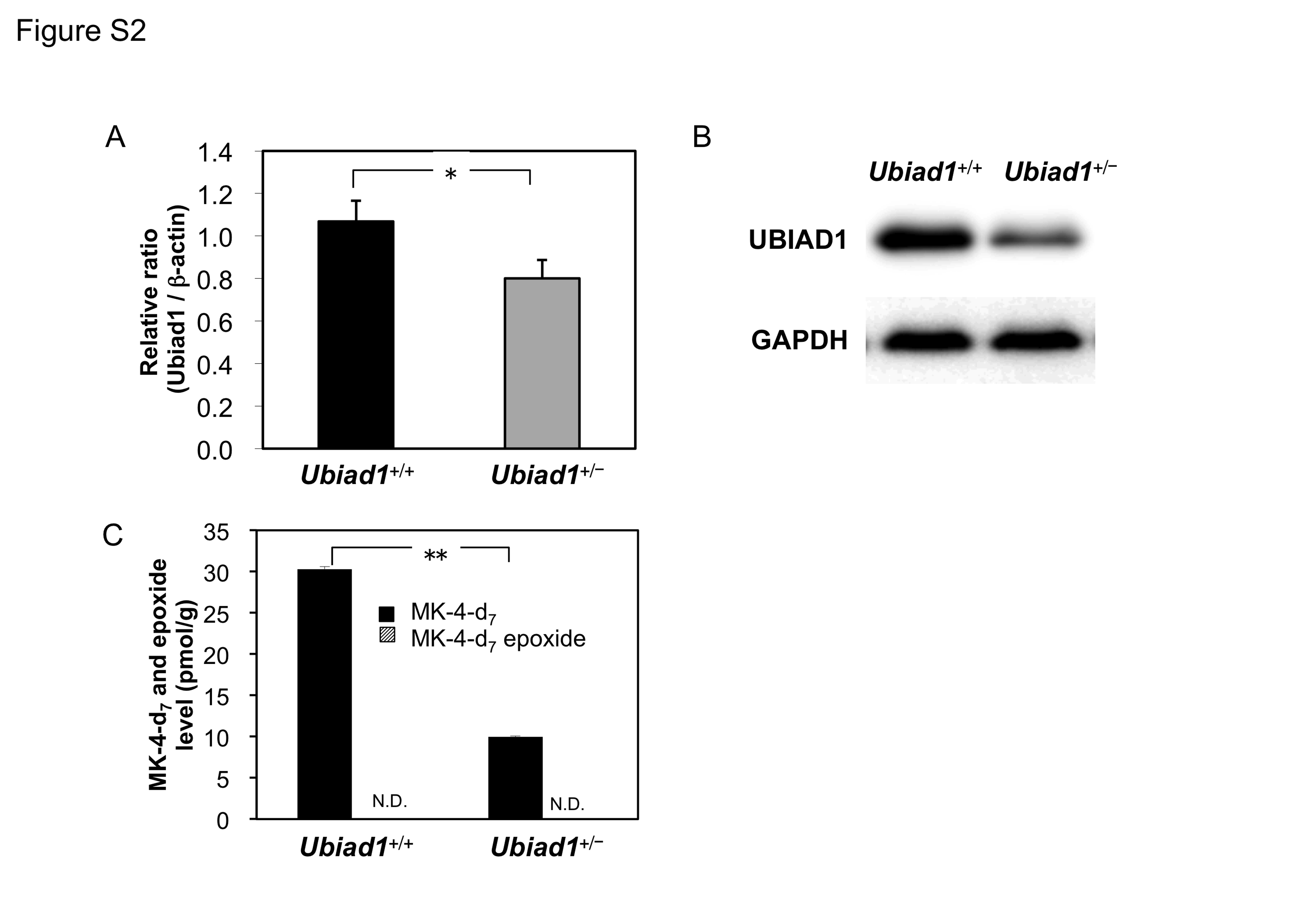

Supplement: Figure S2 — Ubiad1 expression and MK-4 synthesis activity in the cerebrum of Ubiad1 +/+ and Ubiad1 +/− mice. (A) Ubiad1 mRNA expression in the cerebrum of Ubiad1 +/+ and Ubiad1 +/− mice (28 weeks old). (B) UBIAD1 protein expression in the cerebrum of Ubiad1 +/+ and Ubiad1 +/− mice (28 weeks old). (C) The biosynthesis of MK-4-d7 from MD-d8 in the cerebrum of Ubiad1 +/+ and Ubiad1 +/− mice (10 weeks old). Mean ± s.e.m. Student's t test, *P<0.05 and **P<0.01. N.D.: not detected. (TIF) [file pone.0104078.s002.tif]

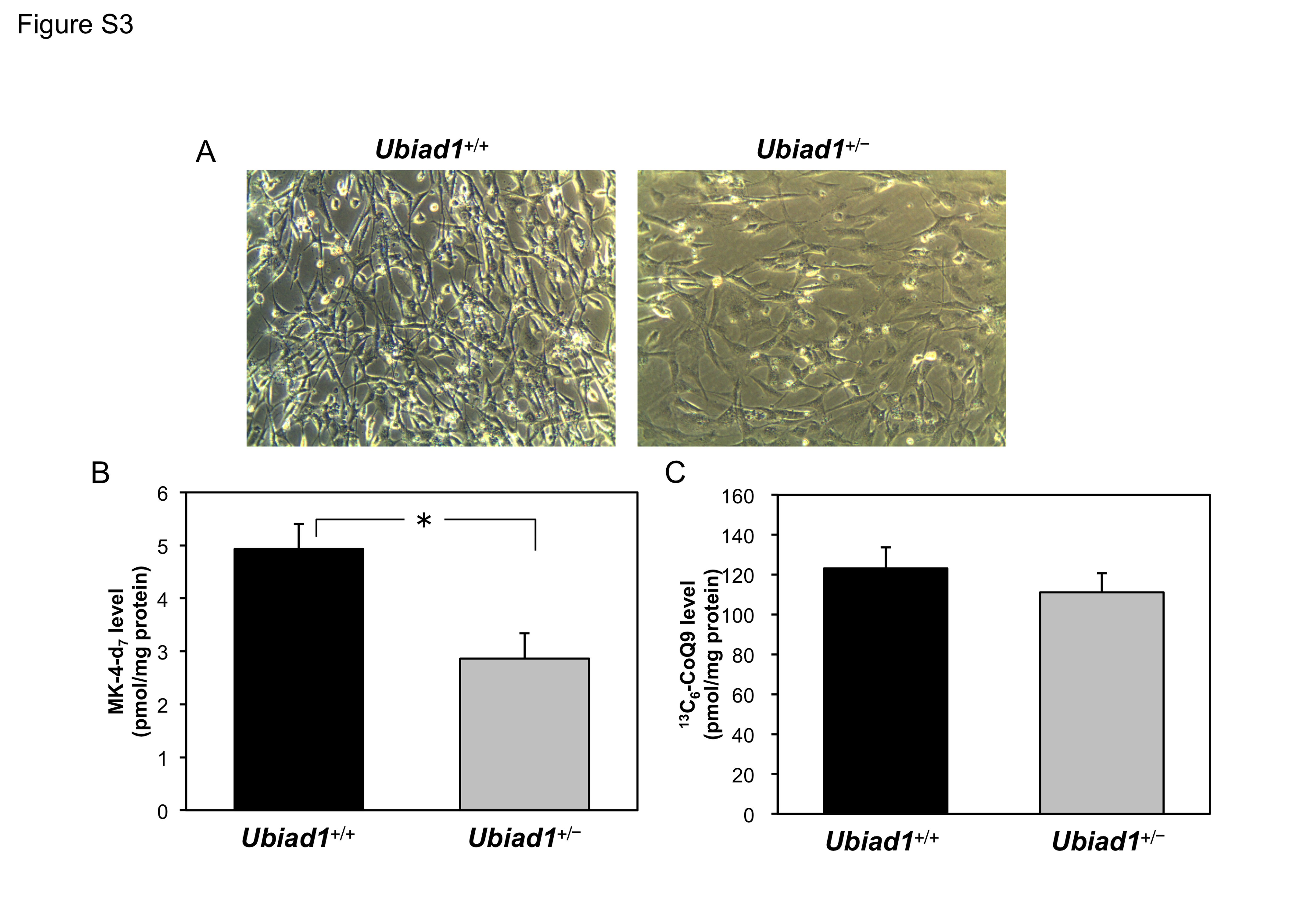

Supplement: Figure S3 — The biosynthesis of MK-4 from MD-d8 and that of CoQ9/CoQ10 from 13C6-4HB in MEF from Ubiad1 +/+ and Ubiad1 +/− mice embryo. (A) Morphology of Ubiad1 +/+ and Ubiad1 +/− MEF cells. (B) The biosynthesis of MK-4-d7 and its epoxide from MD-d8 in MEF cells. (C) The biosynthesis of 13C6-CoQ9 and 13C6-CoQ10 from 13C6-4HB in MEF cells. Mean ± s.e.m. N.D.: not detected. * P<0.05. (TIF) [file pone.0104078.s003.tif]
