# Supplementary material for: Vitamin K2 Biosynthetic Enzyme, UBIAD1 Is Essential for Embryonic Development of Mice
Source: PLoS One. 2014 Aug 15;9(8):e104078. doi: 10.1371/journal.pone.0104078 (PMC4134213; doi:10.1371/journal.pone.0104078)
Supplement: Table S1 — Concentrations of PK, MK-4 and their epoxides in the tissues of Ubiad1 +/+ and Ubiad1 +/− mice (28 weeks old). (DOCX) [file pone.0104078.s004.docx]

Table S1. Concentrations of PK, MK-4 and their epoxides in the tissues of *Ubiad1*^+/+^ and *Ubiad1*^+/-^ mice (28 weeks old)

|  |  |  |  |  |  |  |  |  |  |  |  |  |
| --- | --- | --- | --- | --- | --- | --- | --- | --- | --- | --- | --- | --- |
|  |  | PK | |  | PK epoxide | |  | MK-4 | |  | MK-4 epoxide | |
|  |  | *Ubiad1* ^+/+^ | *Ubiad1* ^+/-^ |  | *Ubiad1* ^+/+^ | *Ubiad1*^+/-^ |  | *Ubiad1* ^+/+^ | *Ubiad1* ^+/-^ |  | *Ubiad1* ^+/+^ | *Ubiad1* ^+/-^ |
|  |  | *pmol/g* | | | | | | | | | | |
|  | Cerebrum | 2.9 ± 0.2 | 2.6 ± 0.1 |  | N.D. | N.D. |  | 505.7 ± 29.4 | 289.9 ± 27.6 | *** | 18.9 ± 1.6 | 18.3 ± 2.5 |
|  | Cerebellum | 12.3 ± 1.3 | 8.4 ±1.2 |  | N.D. | N.D. |  | 1093.9 ± 86.6 | 784.3 ± 61.8 | ** | N.D. | N.D. |
|  | Medulla oblongata | 25.3 ± 12.7 | 20.4 ±2.0 |  | N.D. | N.D. |  | 474.2 ± 32.3 | 300.9 ± 19.4 | ** | N.D. | N.D. |
|  | Olfactory bulb | 27.7 ± 9.3 | 25.0 ± 5.7 |  | N.D. | N.D. |  | 484.6 ± 27.0 | 355.9 ± 27.3 | ** | N.D. | N.D. |
|  | Eye | 12.0 ± 6.0 | 7.3 ± 3.6 |  | N.D. | N.D. |  | 170.1 ± 8.9 | 123.6 ± 7.5 | ** | N.D. | N.D. |
|  | Thyroid gland | 5.9 ± 0.6 | 5.5 ± 0.7 |  | 2.7 ± 0.2 | 2.5 ± 0.3 |  | 2537.1 ± 101.8 | 1583.1 ± 90.1 | *** | 336.9 ± 27.0 | 257.6 ± 11.7 |
|  | Parathyroid gland | 17.7 ± 8.9 | 62.1 ± 31.0 |  | N.D. | N.D. |  | 704.6 ± 72.0 | 432.8 ± 177.0 | * | N.D. | N.D. |
|  | Aorta | 32.0 ± 6.6 | 42.9 ± 7.4 |  | N.D. | N.D. |  | 531.5 ± 66.3 | 451.2 ± 20.3 | ** | 57.6 ± 32.4 | 24.7 ± 18.5 |
|  | Heart | 4.9 ± 2.4 | 4.2 ± 2.1 |  | N.D. | N.D. |  | 308.1 ± 22.9 | 188.1 ± 10.8 | ** | N.D. | N.D. |
|  | Thymus | 10.6 ± 5.3 | 9.0 ± 4.5 |  | N.D. | N.D. |  | 525.9 ± 35.9 | 319.7 ± 15.9 | ** | 103.9 ± 4.5 | 29.2 ± 16.9 |
|  | Lung | 4.6 ± 0.9 | 3.8 ± 0.5 |  | 2.1 ± 0.4 | 1.7 ± 0.2 |  | 100.2 ± 10.0 | 51.6 ± 3.0 | ** | 28.9 ± 1.9 | N.D. |
|  | Liver | 3.2 ± 1.6 | 4.7 ± 3.3 |  | N.D. | N.D. |  | 52.7 ± 3.5 | 34.8 ± 3.1 | * | 12.2 ± 3.4 | N.D. |
|  | Pancreas | 2.8 ± 1.0 | 2.2 ± 0.7 |  | N.D. | N.D. |  | 1761.5 ± 112.8 | 1304.2 ± 113.7 | ** | 444.0 ± 25.3 | 362.3 ± 51.4 |
|  | Spleen | 5.5 ± 0.7 | 5.0 ± 0.7 |  | 2.5 ± 0.3 | 2.3 ± 0.3 |  | 170.9 ± 5.1 | 140.9 ± 26.1 | * | 26.6 ± 2.4 | 23.3 ± 2.6 |
|  | Kidney | 2.1 ± 1.1 | 1.9 ± 0.9 |  | N.D. | 0.5 ± 0.2 |  | 580.3 ± 95.7 | 313.5 ± 16.3 | ** | 30.6 ± 4.1 | 18.8 ± 0.7 |
|  | Adrenal gland | 52.0 ± 12.9 | 49.8 ± 6.8 |  | 23.4 ± 5.8 | 22.4 ± 3.1 |  | 569.3 ± 50.5 | 289.7 ± 45.2 | *** | 34.3 ± 34.3 | N.D. |
|  | Stomach | 10.4 ± 5.2 | 10.3 ± 5.1 |  | N.D. | N.D. |  | 280.3 ± 14.4 | 165.3 ± 13.8 | ** | 25.9 ± 1.0 | 18.8 ± 1.8 |
|  | Duodenum | 5.0 ± 2.5 | 5.8 ± 2.9 |  | N.D. | N.D. |  | 377.6 ± 60.4 | 225.7 ± 28.3 | ** | 79.3 ± 15.2 | 43.9 ± 9.4 |
|  | Small intestine | 23.2 ± 11.6 | 31.0 ± 15.5 |  | N.D. | N.D. |  | 606.3 ± 110.6 | 335.5 ± 44.9 | *** | 25.3 ± 2.3 | 61.8 ± 21.2 |
|  | Large intestine | 2.0 ± 1.0 | 1.0 ± 0.5 |  | N.D. | N.D. |  | 122.8 ± 17.6 | 92.4 ± 17.8 | * | 25.3 ± 2.3 | N.D. |
|  | Musculus | 2.0 ± 1.0 | 1.7 ± 0.9 |  | N.D. | N.D. |  | 149.1 ± 10.0 | 81.5 ± 8.3 | ** | 4.6 ± 4.6 | N.D. |
|  | Ovary | 8.2 ± 4.1 | 4.2 ± 2.1 |  | N.D. | N.D. |  | 373.7 ± 31.6 | 215.5 ± 23.8 | ** | 130.8 ± 16.8 | 59.6 ± 9.8 |
|  | Uterus | 30.6 ± 2.3 | 33.1 ± 3.3 |  | 13.8 ± 1.1 | 14.9 ± 1.5 |  | 761.6 ± 114.8 | 620.4 ± 101.3 |  | 164.8 ± 30.4 | 72.1 ± 42.7 |

N.D.: not detected

Significantly different from ubiad1+/+ mice: * P<0.05, **P<0.01 and ***P<0.001
