# Supplementary material for: Vitamin K2 Biosynthetic Enzyme, UBIAD1 Is Essential for Embryonic Development of Mice
Source: PLoS One. 2014 Aug 15;9(8):e104078. doi: 10.1371/journal.pone.0104078 (PMC4134213; doi:10.1371/journal.pone.0104078)
Supplement: Table S2 — Concentrations of CoQ9 and CoQ10 in the tissues of Ubiad1 +/+ and Ubiad1 +/− mice (28 weeks old). (DOCX) [file pone.0104078.s005.docx]

|  |  |  |  |  | |  |  |
| --- | --- | --- | --- | --- | --- | --- | --- |
|  | CoQ9 | |  | CoQ10 | | |  |
|  | *Ubiad1*^+/+^ | *Ubiad1*^+/-^ |  | *Ubiad1*^+/+^ | | *Ubiad1*^+/-^ |  |
|  | *pmol/mg* | | | | | |  |
| Cerebrum | 0.19 ± 0.1 | 1.33 ± 0.68 | ^NS^ | | 0.15 ± 0.07 | 0.64 ± 0.30 | ^NS^ |
| Heart | 65.92 ± 4.85 | 71.76 ± 5.31 | ^NS^ | | 8.01 ± 0.49 | 7.02 ± 0.68 | ^NS^ |
| Liver | 6.45 ± 3.09 | 4.52 ± 1.04 | ^NS^ | | 0.40 ± 0.26 | 0.06 ± 0.02 | ^NS^ |
| Kidney | 75.22 ± 6.04 | 79.30 ± 8.10 | ^NS^ | | 7.98 ± 1.80 | 8.66 ± 1.02 | ^NS^ |
| Small intestine | 19.45 ± 2.63 | 14.75 ± 5.45 | ^NS^ | | 2.46 ± 0.67 | 2.88 ± 0.65 | ^NS^ |

Table S2. Concentrations of CoQ9 and CoQ10 in the tissues of *Ubiad1*^+/+^ and *Ubiad1*^+/-^ mice (28 weeks old)

NS: not significant difference
