# Supplementary material for: Vitamin K2 Biosynthetic Enzyme, UBIAD1 Is Essential for Embryonic Development of Mice
Source: PLoS One. 2014 Aug 15;9(8):e104078. doi: 10.1371/journal.pone.0104078 (PMC4134213; doi:10.1371/journal.pone.0104078)
Supplement: Table S3 — Blood chemical values of Ubiad1 +/+ and Ubiad1 +/− mice (28 weeks old). (DOCX) [file pone.0104078.s006.docx]

Table S3. Blood chemical values of *Ubiad1*^+/+^ and *Ubiad1*^+/–^ mice (28 weeks old)

|  | Ca | Pi | Glucose | Total Cho | Free-Cho | HDL-Cho | LDL-Cho | Triglyceride |
| --- | --- | --- | --- | --- | --- | --- | --- | --- |
|  | (mg/100 mL) | (mg/100 mL) | (mg/100 mL) | (mg/100 mL) | (mg/100 mL) | (mg/100 mL) | (mg/100 mL) | (mg/100 mL) |
| *Ubiad1*^+/+^ | 8.0 ± 0.32 | 7.3 ± 0.44 | 192.5 ± 14.94 | 63.3 ± 3.40 | 17.8 ± 0.51 | 44.1 ± 2.72 | 7.5 ± 0.48 | 38.8 ± 2.08 |
| *Ubiad*1^+/–^ | 7.9 ± 0.33 | 6.9 ± 0.20 | 181.9 ± 12.27 | 72.6 ± 2.97* | 20.8 ± 0.97* | 59.8 ± 1.40** | 7.2 ± 1.67 | 41.1 ± 2.95 |

Significantly different from *Ubiad1^+/+^* mice: * P<0.05 and **P<0.01
